# Clinical Evaluation and Systematic Classification of Endoscopic Gastrointestinal Findings in 176 French Bulldogs with Brachycephalic Airway Obstructive Syndrome

**DOI:** 10.3390/ani15142137

**Published:** 2025-07-19

**Authors:** Enrico Bottero, Pietro Ruggiero, Daniele Falcioni, Fabiano Raponi, Andrea Campanile, Giuseppe De Cata, Davide De Lorenzi, Samuele Gonella, Emanuele Mussi, Antonio Borrelli, Ugo Ala, Paola Gianella

**Affiliations:** 1 Associazione Professionale Endovet, 00149 Rome, Italy; ruggendo@gmail.com (P.R.); falk@libero.it (D.F.); raponi.fabiano@gmail.com (F.R.); andreacampanile@hotmail.it (A.C.); giuseppedecata82@gmail.com (G.D.C.); samuele.gonella1997@gmail.com (S.G.); e.mussi@yahoo.it (E.M.); 2 San Marco Veterinary Clinic and Laboratory, 35030 Padua, Italy; delorenzi.davide@alice.it; 3 Department of Veterinary Sciences, University of Turin, 10095 Grugliasco, Italy; antonio.borrelli@unito.it (A.B.); ugo.ala@unito.it (U.A.)

**Keywords:** BAOS, brachycephalic breed, dog, endoscopy, gastrointestinal abnormalities

## Abstract

The respiratory consequences of brachycephalic airway obstructive syndrome (BAOS) are well known; however, brachycephalic dogs may also present with alimentary tract signs. The electronic medical records of 176 French bulldogs with BAOS were reviewed to classify the gastrointestinal endoscopic findings, and to evaluate the associations between clinicopathological data, endoscopic respiratory, and digestive findings. Dogs that did not undergo endoscopic examination of both airways and the upper digestive tract were excluded. The type and frequency of respiratory and digestive signs were assessed according to a previously described grading system, in addition to gastrointestinal histopathological findings. Video documentation was reviewed to assign a score to each gastrointestinal endoscopic finding (EGF) and to obtain a total EGF score. All dogs showed at least one EGF. The median total EGF score was 5 (range 1–9). A significant association between the score from digestive signs and the total EGF score was found. In addition, laryngeal granulomas were significantly associated with regurgitation. No associations were found between gastrointestinal histopathological findings and the scores from respiratory or digestive signs. Overall, gastrointestinal endoscopic findings and laryngeal granulomas are common among French bulldogs with BAOS. Therefore, a systematic endoscopic approach to alimentary signs is desirable to determine the most appropriate treatment.

## Simple Summary

This study described the presence and severity of gastrointestinal endoscopic findings in French bulldogs with upper airway obstruction syndrome and evaluated possible associations between digestive signs and other clinicopathological data. Researchers worked with 176 electronic medical records and used validated scores to assess respiratory and digestive signs. In addition, a summative score from individual gastrointestinal endoscopic abnormalities was used. The results showed that gastrointestinal endoscopic abnormalities are very common among French bulldogs with brachycephalic airway obstructive syndrome, and the more severe the digestive signs, the more severe the gastrointestinal endoscopic lesions. The systematic evaluation of gastrointestinal endoscopic abnormalities is easy and safe to perform and adds valuable information for the clinical management of French bulldogs with upper airway obstructive syndrome.

## 1. Introduction

Brachycephalic airway obstructive syndrome (BAOS) is a conformation-related condition that obstructs the upper airways and causes respiratory signs in dog breeds with shortened skulls and muzzles. Affected dogs show noisy and labored breathing, exercise intolerance, sleep disturbed breathing, cyanosis, collapse and death, in addition to a variety of gastrointestinal signs related to upper airway anatomic abnormalities [1,2]. Several studies conducted over the past 15 years have shown that regurgitation and vomiting are the most common gastrointestinal signs associated with this syndrome [1,3,4,5,6]. It is also well established that there are variations in the anatomy, pathophysiology, and clinical presentation of brachycephalic breeds [7,8,9]. For instance, French bulldogs exhibit more severe digestive signs that occur more often than what is observed in pugs [1,5,9,10], with regurgitation being very common, likely due to the high prevalence of hiatal hernia [11]. However, while endoscopic examination is imperative to assess the airways and digestive anatomic abnormalities in brachycephalic breeds [12,13], fluoroscopic examination is recommended for investigating gastroesophageal junction abnormalities, such as gastroesophageal reflux, sliding hiatal hernia, or cardial atony [1,10,11,14,15,16]. Indeed, because of the dynamic nature of gastroesophageal junction abnormalities in brachycephalic breeds, their prevalence based on endoscopic evaluation is likely underestimated [11,17].

Currently, there is no standardized method for evaluating the presence and severity of the gastrointestinal endoscopic findings in dogs with BAOS. Therefore, this retrospective study aimed to outline and classify the gastrointestinal endoscopic findings in a population of French bulldogs with BAOS, and to evaluate possible associations between clinical data, gastrointestinal findings, and respiratory endoscopic findings.

## 2. Materials and Methods

### 2.1. Study Design and Ethics Approval

This was a retrospective investigation that involved client-owned dogs. The experimental protocol was reviewed and approved by the Ethics and Animal Welfare Committee of the University of Turin (protocol number 0000282), and all owners provided informed consent.

### 2.2. Animals

The medical records of client-owned French bulldogs with upper respiratory clinical signs related to BAOS that were referred to Endovet clinics in Northern and Central Italy between January 2021 and January 2023 were retrospectively reviewed. Dogs that did not undergo complete physical examination and systematic endoscopic examination of both the upper and lower respiratory airways and the upper digestive tract were excluded. The information collected included age, sex, body weight, body condition score [BCS], type of diet at the time of referral, comorbidities, and abdominal ultrasound findings. For the evaluation of BCS, a Royal CaninTM 5-point scale was used (1 = very thin, 2 = underweight, 3 = ideal weight, 4 = overweight, 5 = obese). The type and frequency of upper respiratory (e.g., snoring, inspiratory effort, stress or exercise intolerance, syncope) and digestive (e.g., ptyalism, regurgitation, vomiting) signs were assessed according to a previously described grading system (Poncet’s classification) [1]. Additional digestive signs such as diarrhea, flatulence, and hyporexia were recorded. The frequency of both respiratory and digestive signs was classified as follows: never, occasionally (less than once monthly), regularly (once weekly), daily (once daily), often (more than once daily), and constantly. Based on the frequency of each respiratory and digestive sign, a global classification of 3 grades was assigned: grade 1 (absent or minimal), grade 2 (moderate), and grade 3 (severe) [1]. For each dog, a final symptomatic score from the Poncet’s classification of both upper respiratory and digestive signs was obtained.

### 2.3. Endoscopic Procedure

A fasting period of 24 h was required. Premedication protocols were decided on a case-by-case basis. Preoxygenation was provided to all dogs that tolerated it for 5 min. They received it before endoscopy by using a face mask. General anesthesia was induced with 2–4 mg/kg propofol IV and maintained by gas anesthesia (isoflurane or sevoflurane in 100% oxygen). All endoscopic procedures were performed in a standardized fashion by 6 of the authors (E.B., P.R., D.F., F.R., A.C., G.D.) [18,19,20]. Both rigid (Hopkins Oblique Rigid Telescope 30° 64,029 BA with diagnostic sheath 10.5 Fr 64,018 VS or operating sheath 14.5 Fr with working channel 5 Fr 67,065 C; Karl Storz ^®^, Tuttlingen, Germany) and flexible (Video Bronchoscope EB-530S, distal diameter 4.9 mm, working channel 2 mm, working length 60 cm; Fujifilm, Tokyo, Japan/Video Gastroscope EG-530N, distal diameter 5.9 mm, working channel 2 mm, working length 110 cm Fujifilm, Tokyo, Japan/Video Gastroscope EG-530WR, distal diameter 9.4 mm, working channel 2.8 mm, working length 110 cm Fujifilm, Tokyo, Japan) endoscopes were used based on the operator’s preferences and the specific anatomical area. Gastrointestinal mucosal samples were collected on a case-by-case basis and submitted for histological evaluation. The diagnostic interpretation of endoscopic specimens was done according to the simplified histopathologic 4-point scale scoring system (0 = normal, 1 = mild, 2 = moderate, 3 = marked histopathologic change), as previously described [21].

After the induction of general anesthesia, all dogs were placed in sternal recumbency for the systemic evaluation of the soft palate, larynx, trachea, bronchi, esophagus, and cardia. During these evaluations, oxygen was delivered through the working channel of the bronchoscope by jet ventilation or by way of flow-by oxygen with the breathing circuit. Following these evaluations, all dogs were intubated and placed in sternal recumbency for the systematic assessment of the nasopharynx and nasal cavities, and then placed in left lateral recumbency for the systematic assessment of the stomach and duodenum.

### 2.4. Classification of Endoscopic Findings

Videotaping was done with a digital video converter (Canopus ADVC110, Canopus, Rekeo, Modena, Italy) connected to a FireWire 800-equipped Mac computer (Apple MacBook Pro-Core i7 processor 2.2 GHz 15.4-inch, Apple, Turin, Italy). Video documentation for each dog was independently reviewed by 2 authors (E.B., P.R.) in a blind, separate fashion to assign a score to each endoscopic gastrointestinal finding (EGF) (1 present/0 absent). A total EGF score (0–10) was obtained by the sum of each EGF score. A discussion to reach a consensus was done only in cases of divergent opinion.

Esophageal findings were classified as present or absent and included alterations compatible with esophageal deviation (dilation of the cervical lumen or tortuous and non-linear esophagus), cardial atony (abnormally open cardia), hiatal hernia (protrusion of the gastric mucosa toward the thorax in the esophageal lumen as viewed from the esophageal position, or protrusion of the fundic mucosa through the hiatus into the caudal thorax as viewed from the gastric retroflex position), and distal esophagitis (pericardial erythematous striae or diffuse pericardial erythema). In addition, distal esophagitis was classified as mild (<50% of the pericardial mucosa with macroscopic alterations, or cranial extension of the macroscopic alterations <5 cm) or severe (>50% of the pericardial mucosa with macroscopic alterations, or cranial extension of the macroscopic alterations >5 cm). Gastrointestinal findings were classified as present or absent and included alterations compatible with gastric inflammation (diffuse or punctiform hyperemia, edema, or erosion/ulceration), gastric stasis (presence of food more than 24 h after fasting), pyloric hypertrophy (obstructive mucosal fold surrounding the pyloric sphincter), antral folds hypertrophy (thickened mucosal folds of the antrum that were resistant to insufflation), duodenal inflammation (hyperemia, friability, granularity, or erosions), and lymphangiectasia (multifocal to diffuse white foci within the mucosa) [22,23]. In addition to the EGF, information regarding the presence of laryngeal granuloma was recorded. The laryngeal granulomas were defined as exophytic, mucosal nodules arising from the vocal cord of the arytenoid cartilage. They were classified as absent or present. Furthermore, they were subclassified as mild (obstruction of the laryngeal aditus <50%) or severe (obstruction of the laryngeal aditus ≥50%). Finally, the BAOS was endoscopically classified by the authors as severe in the case of stage 3 laryngeal collapse [24,25], or in the presence of at least 4 of the following anatomic alterations: elongated and thickened soft palate, stage 2 of laryngeal collapse, tracheal hypoplasia, stenotic nares, ectopic cranial and caudal turbinates, or intranasal mucosal contact points. On the contrary, the BAOS was endoscopically classified as mild in the of presence of ≤3 of the anatomic alterations.

### 2.5. Statistical Analysis

All statistical analyses were performed using R (version 4.4.0) and RStudio (Build 524). Values of *p* < 0.05 were considered significant. Quantitative data were reported as the median, minimum, and maximum; categorical data were reported as percentages. The Fisher’s exact test was used to study the associations of selected categorical variables, such as the final symptomatic score from the Poncet’s classification of both respiratory and digestive signs, regurgitation, distal esophagitis, hiatal hernia, laryngeal granuloma, total EGF score, BAOS severity, and the intestinal simplified histopathologic scoring system results. In addition, the Kruskal–Wallis test was applied to analyze the total EGF as a function of the symptomatic score from the Poncet’s classification of digestive signs. Subsequent pairwise comparisons using the Wilcoxon rank sum test with continuity correction were also performed, when possible, through the pairwise.wilcox.test function, with the false discovery rate (FDR) applied for the *p*-value adjustment.

## 3. Results

### 3.1. Patient Data

The final study population consisted of 176 French bulldogs with BAOS. Among them, 133 (75.6%) were male (5 neutered) and 43 (24.4%) were female (19 spayed). The median age was 24 months (range 2–120), the median body weight was 12 kg (range 5.7–24), and the median BCS was 3 (range 2–5). A BCS of 2 was assigned to 13 (7.4%) dogs, a BCS of 3 to 114 (64.8%) dogs, a BCS of 4 to 48 (27.3%) dogs, and a BCS of 5 to 1 (0.5%) dog. Respiratory signs were classified as grade 1 in 37 (21.1%) dogs, grade 2 in 96 (54.5%) dogs, and grade 3 in 43 (24.4%) dogs. Digestive signs were classified as grade 1 in 53 (30.1%) dogs, grade 2 in 70 (39.8%) dogs, and grade 3 in 53 (30.1%) dogs. In addition to the digestive signs from the Poncet’s classification, diarrhea, flatulence, and hyporexia were reported in 33 (18.7%), 2 (1.1%), and 1 (0.6%) dog, respectively. Overall, the most prevalent gastrointestinal signs were regurgitation (82 dogs, 46.6%), vomiting (22 dogs, 12.5%), ptyalism (21 dogs, 11.9%), and diarrhea (10 dogs, 5.7%). Forty-one (23.3%) dogs showed no prevalent digestive signs. Among the 43 dogs that were classified as grade 3 from the Poncet’s classification of upper respiratory signs, 19 (44.2%) were classified as grade 3 from the Poncet’s classification of digestive signs.

At the time of diagnosis, 159 (90.3%) dogs were fed commercial diets of different types and brands, while 17 (9.7%) dogs were fed home-cooked diets. Specifically, 80 (50.3%) dogs were on nutritionally complete and balanced commercial diets, 37 (23.3%) were on limited-ingredient commercial diets, 30 (18.9%) were on highly digestible gastrointestinal commercial diets, and 12 (7.5%) were on hydrolyzed commercial diets. Comorbidities were identified in 27 (15.3%) dogs, of which 19 (70.4%) had dermatopathies, 5 (18.5%) had orthopedic disorders, and 3 (11.1%) had neuropathies.

Abdominal ultrasounds were performed in 87 (49.4%) dogs, 21 of which had diarrhea. Of these 87 dogs, alterations to the mucosal echogenicity (hyperechoic speckles) were found in 38 (43.7%) dogs, 15 of which had diarrhea, while pyloric hypertrophy and prominent gastric mucosal folds were found in 2 (2.3%) dogs. Enlarged abdominal lymph nodes, altered intestinal stratigraphy/thickness, and altered hepatic echogenicity or hepatomegaly were not found. No abnormal gastrointestinal findings were observed in the remaining 47 (54%) dogs, 6 of which had diarrhea.

### 3.2. Endoscopic Classification of the Gastrointestinal and Respiratory Findings

**Figure 1:**
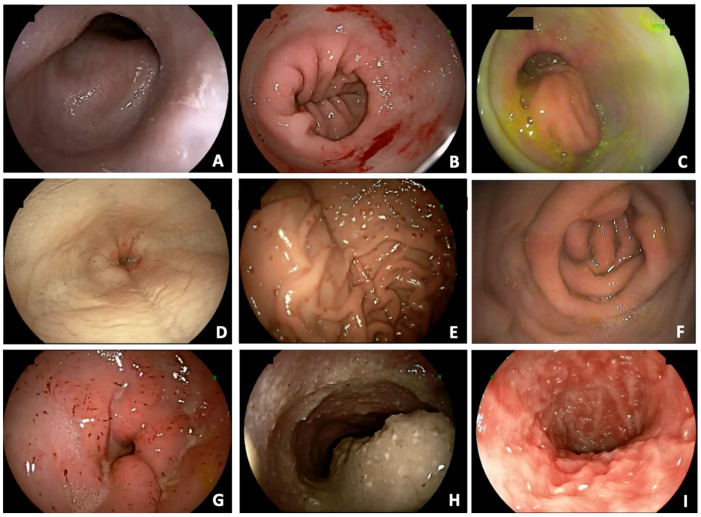
Gastrointestinal endoscopic findings in French bulldogs with BAOS. (**A**) Esophageal deviation; (**B**) cardial atony; (**C**) hiatal hernia; (**D**) distal esophagitis; (**E**) gastric inflammation; (**F**) antral fold hypertrophy; (**G**) pyloric hypertrophy; (**H**) gastric stasis; (**I**) duodenal inflammation and lymphangiectasia.

Esophageal deviation (88.1%), distal esophagitis (79%), cardial atony (29%), and hiatal hernia (26.7%) were observed in 155, 139, 51, and 47 dogs, respectively. Distal esophagitis was considered mild in 88 (63.3%) dogs and severe in 51 (36.7%) dogs. Overall, of the 139 dogs with distal esophagitis, 62 (44.6%) did not show regurgitation. On the contrary, 7 dogs without endoscopic alterations compatible with esophagitis showed regurgitation. Alterations compatible with gastric inflammation (77.3%), pyloric hypertrophy (21%), antral fold hypertrophy (39.2%), and gastric stasis (21.6%) were observed in 136, 37, 69, and 38 dogs, respectively. Alterations compatible with duodenal inflammation (69.9%) and lymphangiectasia (34.1%) were observed in 123 and 60 dogs, respectively. Examples of the gastrointestinal endoscopic findings are shown in Figure 1.

Overall, all dogs showed at least one gastrointestinal endoscopic alteration. Specifically, 115 (65.3%) dogs showed endoscopic alterations of all three organs (esophagus, stomach, and duodenum), 170 (96.6%) dogs showed at least one esophageal alteration, 154 (87.5%) dogs showed at least one gastric alteration, and 123 (69.9%) dogs showed at least one duodenal alteration. Among esophageal alterations, 27 (15.3%), 81 (46%), 45 (25.6%), and 17 (9.7%) dogs showed one, two, three, and four out of four esophageal alterations, respectively. Six (3.4%) dogs did not show any esophageal alteration. Among gastric alterations, 70 (39.8%), 52 (29.5%), 22 (12.5%), and 10 (5.7%) dogs showed one, two, three, and four out of four gastric alterations. Twenty-two (12.5%) dogs did not show gastric alterations. Among duodenal alterations, 63 (35.8%) and 60 (34.1%) dogs showed one and two out of two duodenal alterations. Fifty-three dogs did not show duodenal alterations. Table 1 and Table 2 summarize the endoscopic gastrointestinal findings.

**Figure 2:**
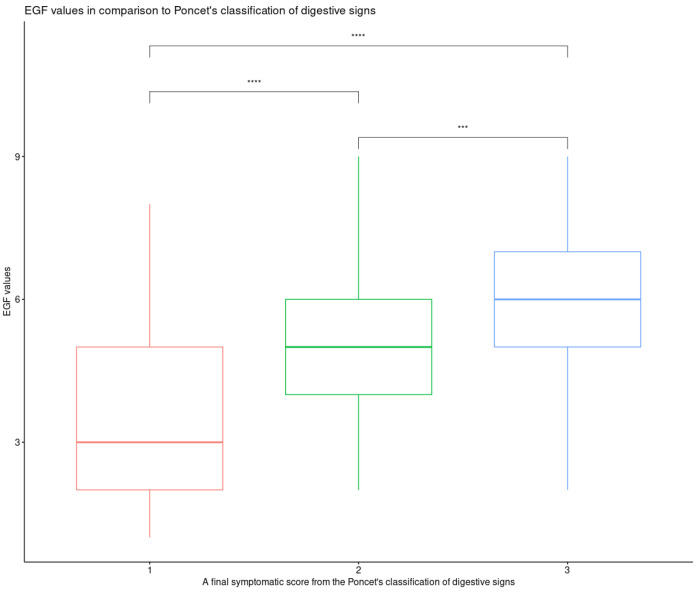
Comparison of EGF scores across three subject groups (Kruskal–Wallis, *p* = 1.7 × 10^−10^), categorized according to the global classification of digestive signs (Poncet’s classification). All pairwise comparisons yielded significant results: group 1 vs. group 2 (*p* = 2.3 × 10^−5^); group 1 vs. group 3 (*p* = 2.6 × 10^−9^); group 2 vs. group 3 (*p* = 0.00021). **** *p* values < 10^−4^; *** *p* values < 10^−3^.

Total EGF scores of 1, 2, 3, 4, 5, 6, 7, 8, and 9 were assigned to 4 (2.3%), 13 (7.4%), 29 (16.5%), 32 (18.2%), 36 (20.5%), 30 (17%), 15 (8.5%), 8 (4.5%), and 9 (5.1%) dogs, respectively. A total EGF score of 10 was not assigned. The median total EGF score was 5 (range 1–9). A significant association between the final symptomatic score from the Poncet’s classification of digestive signs and the total EGF (*p* = 0.0005) score was found. Particularly, the median value of the total EGF score for dogs classified as grade 3 was significantly higher than that for dogs classified as grade 2 and 1, respectively (*p* = 1.7 × 10^−5^) (Figure 2).

A significant association between the final symptomatic score from the Poncet’s classification of upper respiratory signs and the total EGF score was not found.

**Figure 3:**
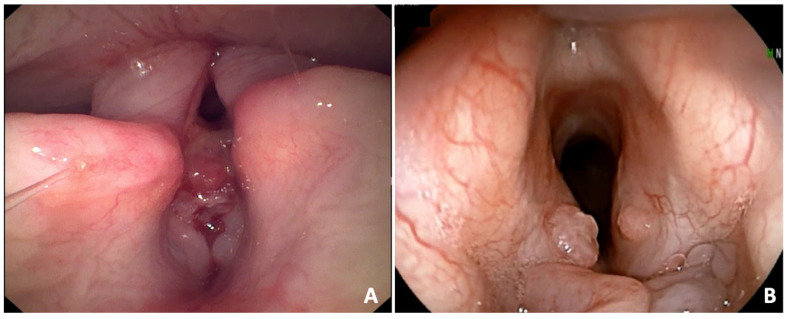
Laryngeal granulomas in French bulldogs with BAOS. (**A**) Severe laryngeal granuloma; (**B**) mild laryngeal granuloma.

Laryngeal granulomas were observed in 56 (31.8%) dogs (Figure 3). They were classified as severe in 10 (17.9%) dogs and mild in 46 (82.1%) dogs. The BAOS was endoscopically classified as severe in 60 (34.1%) dogs and mild in 116 (65.9%) dogs. A significant association between BAOS severity and the final symptomatic score from the Poncet’s classification of digestive signs was found (*p* = 0.016). No significant associations between laryngeal granuloma and hiatal hernia or esophagitis were found. A significant association between laryngeal granuloma and regurgitation was found (*p* = 0.014). No significant associations between the endoscopic BAOS severity and the final symptomatic score from the Poncet’s classification of upper respiratory signs and the total EGF score were found.

### 3.3. Histopathological Findings

A histopathological evaluation of the gastrointestinal mucosal samples was available for 56 (31.8%) dogs. A lymphoplasmacytic infiltration of the gastric and intestinal mucosae was found in 27 (48.2%) and 32 (57.1%) dogs, respectively. Of these dogs, 30 had diarrhea. In addition, gastric subepithelial edema and hemorrhages were found in 18 (32.1%) and 4 (7.1%) dogs, respectively. Regarding the histologic lesion severity, mild (grade 1) duodenal histologic lesions were not found, moderate (grade 2) duodenal histologic lesions were found in 10 (17.9%) dogs, and marked (grade 3) duodenal histologic lesions were found in 46 (82.1%) dogs. Lacteal dilation was found in 17 (30.3%) dogs, 11 of which had diarrhea. No abnormal histopathological findings were found in three dogs with diarrhea. No significant associations were found between the simplified histopathologic scoring system and the final symptomatic score from the Poncet’s classification of both upper respiratory and digestive signs, the total EGF score, and the endoscopic BAOS severity.

## 4. Discussion

This retrospective study aimed to outline and classify the gastrointestinal endoscopic findings in a population of French bulldogs with BAOS, and to investigate possible associations between clinical data, gastrointestinal endoscopic findings, and selected respiratory endoscopic findings.

Over the past 15 years, there has been a significant increase in the identification of aerodigestive disorders, especially in brachycephalic dogs [7]. Among these, French bulldogs seem to be the most frequently and severely affected by gastrointestinal symptoms [1,5,10]. Indeed, 93% of French bulldogs showed severe gastrointestinal signs (grade 2 or 3 according to Poncet’s classification) compared to 58% and 16% of English bulldogs and pugs, respectively [1,5]. Furthermore, a relationship between the severity of respiratory and digestive signs was found to be significant in heavy and male French bulldogs [1,5,10]. According to these observations, 75.6% of our dogs were male and showed severe digestive signs (grade 2 and 3, according to Poncet’s classification). In addition, both severe digestive and respiratory signs (grade 3, according to Poncet’s classification) were observed in just under half of the dogs (44.2%). Overall, the most prevalent gastrointestinal sign was regurgitation (46.6%). The link between regurgitation and BAOS is believed to be associated with an increase in intrathoracic negative pressure, leading to gastroesophageal reflux, hiatal hernia, and esophagitis, as confirmed by experimental studies [26]. However, 44.6% of dogs included in this study with distal esophagitis did not show regurgitation. This is not surprising, and may be due to underreporting by owners, individual variability in symptom expression, or endoscopic examination before the onset of signs. On the contrary, some dogs without endoscopic changes consistent with esophagitis did show regurgitation. However, this result may be due to antiacid medications or other esophageal issues, such as esophageal deviation or dysmotility [27,28]. Finally, a non-erosive reflux disease, as described in human medicine, cannot be ruled out [29,30]. In addition to the gastrointestinal signs of the Poncet’s classification, diarrhea was observed in 18.5% of dogs, and in 5.7% of them it was the prevalent digestive sign. At the time of diagnosis, all dogs with diarrhea were on different commercial or home-cooked diets specifically labeled or formulated for gastrointestinal problems. A minority of dogs with diarrhea did not show abnormalities either on an abdominal ultrasound or during a histopathological evaluation. However, most of them had macro- and microscopic findings compatible with chronic gastrointestinal inflammation. Overall, gastritis and duodenitis were diagnosed in 27 and 32 out of 56 dogs that underwent histopathological evaluation of gastrointestinal mucosal samples, respectively, while lymphangiectasia was found in 17 dogs. All these dogs showed endoscopic findings suggestive of inflammation; however, vomiting and diarrhea were the prevalent sign in only a minority of these dogs. The high prevalence of macroscopic gastrointestinal alterations is not surprising, as inflammation can be diagnosed in 26–48% of asymptomatic dogs [31,32]. The continuous swallowing of air may have contributed to the pathogenesis of chronic intestinal inflammation [31,32]. However, the presence of concurrent food- or immunosuppressant-responsive enteropathies may also have contributed. Indeed, a relationship among the severity of clinical signs, the severity of histopathological findings, and endoscopic alterations of the digestive system has been hypothesized. However, this relationship has not yet been fully understood [1,33].

Currently, a standardized endoscopic classification of the gastrointestinal findings in brachycephalic dogs is lacking. In this study, to minimize errors stemming from interpretative subjectivity, the endoscopic evaluation of the gastrointestinal system was condensed into an overall endoscopic score (total EGF). As expected, a significant association was found between this score and the final symptomatic score from the Poncet’s classification of digestive signs. However, in veterinary literature, only endoscopic evaluations of single gastrointestinal abnormalities are available, such as the obstruction maneuver during endoscopy for the detection of dynamic gastro-esophageal junction abnormalities [17,34]. In our study, the esophagus was evaluated before intubation, allowing for the inspection of both esophageal and gastric side of the cardias. Since this maneuver is rapid, no complications occurred. However, a careful approach to avoid larynx compression and overinflation of the stomach is needed. Gastroesophageal reflux was not considered in the total EGF. Indeed, its presence/absence is difficult to define, and it would be inaccurate to assess it as absent only because it was not observed during the few minutes of endoscopic evaluation, as previously stated [17]. Instead, the presence/absence and severity of distal esophagitis were evaluated, since they have a link with gastroesophageal reflux and hiatal hernia [35]. However, 7 out of 47 dogs with hiatal hernia did not have endoscopic alterations compatible with distal esophagitis. This result could be due to recent antacid therapy or mild esophagitis without endoscopically identifiable lesions. However, a false positive endoscopic result due to the anesthesia causing an increased and abnormal laxity of the esophageal hiatus cannot be ruled out. The hiatal hernia was evaluated here using the definition previously reported [17,34,35], although the authors believe that fluoroscopy is a more effective method [11,14,36]. Pyloric hypertrophy and antral folds hypertrophy were relatively common endoscopic findings in dogs included in this study. However, since no clinical correlation with vomiting or regurgitation was found, it can be assumed that the abnormal endoscopic appearance of the pyloric antrum in brachycephalic dogs, although frequent, lacks clinical significance. No significant associations between regurgitation, presence of endoscopic alterations compatible with esophagitis, and hiatal hernia were found. Instead, a significant association between laryngeal granuloma and regurgitation was found. Laryngeal granulomas are rarely observed in brachycephalic breeds, but they are often reported in humans as contact granulomas [37,38]. In humans, granulomas result from chronic physical or chemical insult to laryngeal mucosa. In brachycephalic breeds, granulomas may result from chronic inspiratory efforts, air turbulence, and gastro-esophageal reflux leading to chronic laryngeal inflammation. In this study, laryngeal granulomas were found in 31.8% of dogs, suggesting that they could be more common than previously reported, at least in French bulldogs with BAOS.

In the veterinary literature, there are descriptions of the individual components of BAOS, such as Leonard’s classification of laryngeal collapse [24], and many authors highlight the usefulness of endoscopic evaluation for BAOS [1,2]. However, a standardized classification of all endoscopic respiratory abnormalities in brachycephalic breeds is lacking. Recently, a more comprehensive evaluation of endoscopic respiratory abnormalities was proposed [13]. In our study, since the primary aim was the systematic evaluation of endoscopic gastrointestinal abnormalities, a simplified and non-validated method for the evaluation of endoscopic airway abnormalities was adopted, assigning the dogs to two different groups based on the endoscopic BAOS severity obtained by the sum of the individual respiratory abnormalities. A significant association between this simplified method of the endoscopic BAOS severity and the final symptomatic score from the Poncet’s classification of digestive signs was found, as previously observed [1]. However, since we used a non-validated respiratory endoscopic score, further assessments would be purely speculative.

This study has several limitations. These include its retrospective nature, the subjective interpretation of endoscopic abnormalities, the use of a non-validated method of endoscopic assessment of BAOS severity, and the absence of gastrointestinal histopathological evaluations for all dogs. Finally, there were a lack of data related to fluoroscopy and respiratory function tests, which allow for a more consistent and accurate classification of digestive and respiratory findings.

## 5. Conclusions

Several upper digestive endoscopic abnormalities were described in French bulldogs using a systematic endoscopic approach and by identifying the presence of laryngeal granulomas. Moreover, a significant association between the severity of digestive signs and gastrointestinal endoscopic findings was found, along with an association between regurgitation and laryngeal granulomas. Overall, the systematic evaluation of upper gastrointestinal endoscopic abnormalities was found to be safe and easy to perform and provided valuable information for the clinical management of French bulldogs with BAOS.

## Figures and Tables

**Table 1:** Gastrointestinal endoscopic findings in 176 French bulldogs with BAOS.

Gastrointestinal Tract	Lesion	Number of Dogs (%)
Esophagus	Esophageal deviation	155 (88.1%)
	Distal esophagitis	139 (79%)
	Cardial atony	51 (29%)
	Hiatal hernia	47 (26.7%)
Stomach	Gastric inflammation	136 (77.3%)
	Pyloric hypertrophy	37 (21%)
	Antral fold hypertrophy	69 (39.2%)
	Gastric stasis	38 (21.6%)
Duodenum	Duodenal inflammation	123 (69.9%)
	Lymphangiectasia	60 (34.1%)

**Table 2:** Number (%) of French bulldogs showing 0 to 4 esophageal and gastric endoscopic findings, and 0 to 2 duodenal endoscopic findings.

	0 *	1 *	2 *	3 *	4 *
Esophagus	6 (3.4%)	27 (15.3%)	81 (46%)	45 (25.6%)	17 (9.7%)
Stomach	22 (12.5%)	70 (39.8%)	52 (29.5%)	22 (12.5%)	10 (5.7%)
Duodenum	53 (30.1%)	63 (35.8%)	60 (34.1%)	-	-

* Number of esophageal, gastric, and duodenal endoscopic findings.

## Data Availability

All data analyzed during this study are included in this published article.

## Abbreviations

The following abbreviations are used in this manuscript:

BAOS	Brachycephalic airway obstructive syndrome
EGF	Endoscopic gastrointestinal findings
